# Editorial for Special Issue: The Chorioallantoic Membrane (CAM) Model—Traditional and State-of-the Art Applications: The 1st International CAM Conference

**DOI:** 10.3390/cancers15030772

**Published:** 2023-01-26

**Authors:** Regine Schneider-Stock, Georg Flügen

**Affiliations:** 1 Experimental Tumorpathology, Institute of Pathology, Universitätsklinikum Erlangen, FAU Erlangen-Nürnberg, 91054 Erlangen, Germany; 2 Comprehensive Cancer Center Erlangen-EMN (CCC ER-EMN), University Hospital Erlangen, Friedrich Alexander University Erlangen-Nürnberg, 94054 Erlangen, Germany; 3 Department of General, Visceral, Thoracic and Pediatric Surgery (A), Medical Faculty, Heinrich-Heine-University, University Hospital Duesseldorf, 40225 Duesseldorf, Germany

## 1. Introduction

In 1959, Russell and Burch published the “Principles of Humane Experimental Technique” [1]. They introduced the 3R principles as the replacement, reduction, or refinement of animal experiments. Over the past 25 years, the 3Rs have gained a general acceptance worldwide and they have been included in all the legislations controlling the use of laboratory animals.

Nevertheless, for a realistic mirror imaging of complex tumor growth features, we require in vivo models. Although 2D cell line experiments are very common in basic and translational research, they can analyze only single tumor biological aspects. The complex 3D tumor structure and the role of the tumor environment can be only marginally simulated and the drug response prediction is very limited, as seen by the high failure rate at the translation from preclinical to clinical trials [2].

In vivo studies refer to the execution of experiments inside a living organism, no matter if this organism is a plant, a microorganism, an animal, or a human. The level of complexity present in these living environments, with a fundamentally different 3D extracellular matrix, different cell–cell interactions, and a dynamic blood flow, is as of yet unattainable in any in vitro setting. Thus, in vivo experiments still comprise the gold standard of biomedical research prior to a clinical setting. Yet, the pain and suffering of experimental animals is a cause for ethical concerns and therefore a 3R (reduce, replace, and refine) approach to in vivo research is clearly warranted.

Here, the chorioallantoic membrane (CAM) assay comes into focus. The avian CAM model, although legally not considered an animal model, is still biologically such an in vivo model. The developing avian embryo and, most importantly, its extraembryonic tissue, the CAM, are comprised of epithelial and mesenchymal cells, an extracellular matrix, and complex vasculature. The circulatory system of the avian embryo supplies nutrients and blood to the CAM, providing an ideal microenvironment to study complex cell–cell and cell–matrix interactions, such as those happening during the metastatic cascade. As further discussed in this *Cancers* Special Issue, the lack of an adaptive immune system of the avian embryo between day ED 10 and day ED 14 allows for the xenotransplantation of tumor cells derived from non-avian organisms (i.e., human and rodents).

**Figure 1:**
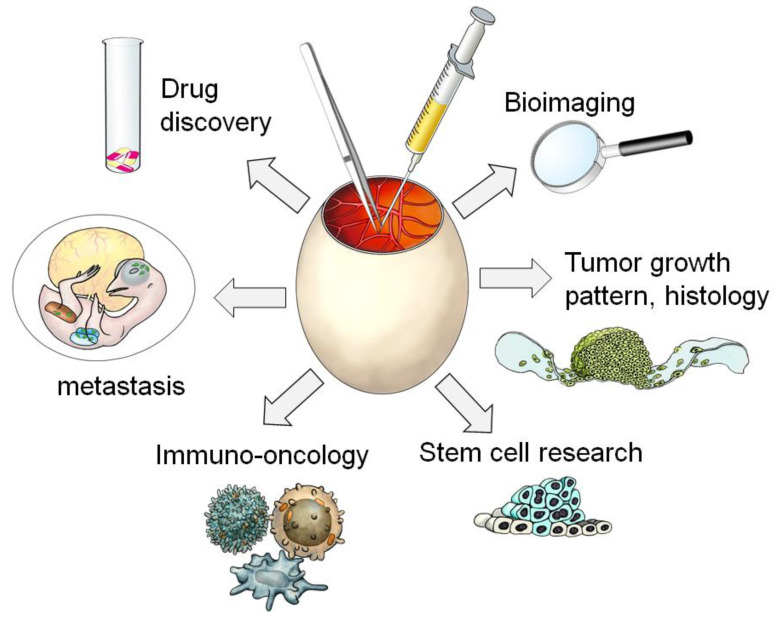
The huge panel of applications for the CAM model [thanks to Jörg Pekarsky (Institute of Functional and Clinical Anatomy, Friedrich Alexander University Erlangen-Nürnberg) for his help with this self-made artwork].

This *Cancers* Issue is a direct result of the first international CAM conference held in February 2022 in a virtual format with more than 300 participants from 36 countries. Researchers from many different disciplines exchanged their knowledge and presented the immense variety of research approaches using the CAM model. The collection of manuscripts in this CAM Issue shows a variety of applications of the CAM model, verifying it as an attractive alternative, a robust, cost effective, and naturally immune-compromised model to study the hallmarks of cancer, such as angiogenesis, proliferation, and tumor invasion, as well as concepts of tumor therapy (Figure 1). 

## 2. Survey for Papers in This Special CAM Issue

Three papers are focusing on the potential of the CAM assay to analyze angiogenesis-associated effects. Demcisakova et al. analyzed the vascularization potential of biomaterial scaffolds that were positioned on the CAM as a test model for novel bone repair materials [3]. Heuberger et al. reported that the potential anti-cancer effect of copper chelators was mostly mediated through angiosuppression [4]. Faihs et al. introduced the application of a novel image analysis software for angiogenesis research [5].

Another set of papers describes the high potential of bioimaging applications. Barnett et al. showed that patient’s mesothelial tumor cell lines can be successfully grown in the CAM model and ovografts were fully recapitulating the different histological subtypes [6]. They suggest that the CAM model will be a suitable pre-clinical in vivo test system for this aggressive tumor type. Moreover, the pre-labeling of tumor cells allowed for longitudinal bioluminescence imaging. In another study, it was reported that radiotracers specifically accumulate in the ovografts and can be followed in the CAM model using positron emission tomography and magnetic resonance scanning [7]. Moreover, the specific target-binding of radiopharmaceuticals was assessed in MRI using specific blocking agents [8]. Consequently, the authors suggested the CAM model as an effective pre-screening assay to detect the bio-distribution of novel radiolabeled compounds saving animal experiments in mice. In another paper, Buschmann et al. showed that a functional gas challenge is feasible on the CAM and allows to non-invasively assess the vascular function and oxygenation phenotype of ovografts [9]. Kunze et al. analyzed invasive colon tumor cells and their potential to remodel the extracellular matrix of the CAM using multiphoton microscopy [10]. Here, the authors take advantage of the ECM composition of the CAM that is a collagen I-rich tissue resembling the structure of many organ tissue layers.

What we need for replacement strategies are more direct comparisons between the CAM and the mouse models for specific applications. In this regard, Rupp et al. treated a large panel of different human and mouse tumor cell lines with clinically approved anti-cancer drugs and demonstrated similar effects in both mice and chicken embryo xenografts [11]. The INOVOTION team described new and impressive applications in the field of immune oncology. Wang et al. showed the anti-cancer effects of PD-L1 immune checkpoint inhibitors in lung cancer cells [12]. They use a prolonged time-window till ED18 to obtain a more effective immune response. For the first time, Rousset et al. successfully isolated circulating tumor cells from cancer patients and engrafted them as patient-derived xenografts (PDX). In their detailed molecular and histological analysis, they discussed potential improvements for this approach to be used for a personalized therapy strategy [13].

The broad spectrum of applications as presented in this *Cancers* issue is primarily based on the simple and direct physical access to the CAM. In particular, this easily accessible assay site will allow for a further standardization and automation of this model’s system to develop more user-friendly applications. The promotion and elaboration of the CAM model presents the major goal the CAM community has to work to achieve.
